# Amelioration of bleomycin-induced pulmonary fibrosis via TGF-β-induced Smad and non-Smad signaling pathways in galectin-9-deficient mice and fibroblast cells

**DOI:** 10.1186/s12929-020-0616-8

**Published:** 2020-01-15

**Authors:** Yu-An Hsu, Ching-Yao Chang, Joung-Liang Lan, Ju-Pi Li, Hui-Ju Lin, Chih-Sheng Chen, Lei Wan, Fu-Tong Liu

**Affiliations:** 1 School of Chinese Medicine, China Medical University, No. 91, Hsueh-Shih Road, Taichung, 40402 Taiwan; 2 Department of Biotechnology, Asia University, Taichung, 40402 Taiwan; 3 Rheumatology Research Center, China Medical University Hospital, Taichung, 40402 Taiwan; 4 School of Medicine, China Medical University, Taichung, 40402 Taiwan; 5 Division of Immunology and Rheumatology, Department of Internal Medicine, China Medical University Hospital, Taichung, 40402 Taiwan; 6 Department of Ophthalmology, China Medical University Hospital, Taichung, 40402 Taiwan; 7 Division of Chinese Traumatology, China Medical University Hospital, Taichung, 40402 Taiwan; 8 Division of Chinese Medicine, Asia University Hospital, Taichung, 40402 Taiwan; 9 Department of Gynecology, China Medical University Hospital, Taichung, 40402 Taiwan; 10 Institute of Biomedical Sciences, Academia Sinica, Taipei, 11529 Taiwan; 11 Department of Dermatology, University of California, Davis, School of Medicine, Sacramento, CA 95816 USA

**Keywords:** Galectin-9, Bleomycin, TGF-β, Fibrosis, Systemic sclerosis

## Abstract

**Background:**

Galectin-9 is a β-galactoside-binding protein with two carbohydrate recognition domains. Recent studies have revealed that galectin-9 regulates cellular biological reactions and plays a pivotal role in fibrosis. The aim of this study was to determine the role of galectin-9 in the pathogenesis of bleomycin-induced systemic sclerosis (SSc).

**Methods:**

Human galectin-9 levels in the serum of patients with SSc and mouse sera galectin-9 levels were measured by a Bio-Plex immunoassay and enzyme-linked immunosorbent assay. Lung fibrosis was induced using bleomycin in galectin-9 wild-type and knockout mice. The effects of galectin-9 on the fibrosis markers and signaling molecules in the mouse lung tissues and primary lung fibroblast cells were assessed with western blotting and quantitative polymerase chain reaction.

**Results:**

Galectin-9 levels in the serum were significantly higher (9-fold) in patients compared to those of healthy individuals. Galectin-9 deficiency in mice prominently ameliorated epithelial proliferation, collagen I accumulation, and α-smooth muscle actin expression. In addition, the galectin-9 knockout mice showed reduced protein expression levels of fibrosis markers such as Smad2/3, connective tissue growth factor, and endothelin-1. Differences between the wild-type and knockout groups were also observed in the AKT, mitogen-activated protein kinase, and c-Jun N-terminal kinase signaling pathways. Galectin-9 deficiency decreased the signal activation induced by transforming growth factor-beta in mouse primary fibroblasts, which plays a critical role in fibroblast activation and aberrant catabolism of the extracellular matrix.

**Conclusions:**

Our findings suggest that lack of galectin-9 protects against bleomycin-induced SSc. Moreover, galectin-9 might be involved in regulating the progression of fibrosis in multiple pathways.

## Background

Systemic sclerosis (SSc) is a chronic systemic connective tissue disease that exhibits characteristics such as essential vasculopathy; fibrosis in the skin, subcutaneous tissue, muscles, and internal organs (e.g., alimentary tract, lungs, heart, kidney, central nervous system); and immunologic activation [1, 2]. The pathophysiology of SSc is unknown, and there are no effective therapies for the disease. Recent studies of the multifaceted etiopathogenesis for the whole disease or organ-specific SSc have revealed numerous molecular targets for potential therapeutic interventions [3]. For many years, activation of fibroblasts was considered to result in excess extracellular matrix deposition in the pathogenesis of SSc. However, recent evidence suggests that the activation of fibroblasts is in fact orchestrated by other cells.

Several potent profibrotic genes were found to be up-regulated in SSc, including transforming growth factor beta (TGF-β), interleukin-4 (IL-4), platelet-derived growth factor, monocyte chemoattractant protein-1, and connective tissue growth factor (CTGF) [4]. TGF-β is expressed in activating fibroblasts and elevates the synthesis of collagen, which is encoded by the *COL1A1* gene. TGF-β also increases proteoglycan synthesis and inhibits extracellular matrix degradation by decreasing matrix metalloproteinase (MMP) synthesis and enhancing tissue inhibitor of MMP expression [5]. TGF-β binds to its receptor TGFβRI to activate its transducing signal into the nucleus via Smad2 and Smad3 phosphorylation. Smad6 and Smad7 are inhibitory Smads that mediate negative feedback by inhibiting TGF-β signaling via forming a complex with Smurf E3 ubiquitin ligase. Moreover, disrupting the functions of Smad3 and Smad7 in SSc reduces the degree of fibrosis [6]. Endothelin-1 (ET-1) and CTGF are produced by endothelial cells and fibroblasts in the early and late phases of SSc. ET-1 is a vasoconstrictor that can stimulate collagen synthesis and inhibit MMP expression, leading to vasculopathy in SSc. CTGF was also observed to be overexpressed in SSc by TGF-β-activated fibroblasts to stimulate collagen production [7, 8].

Galectin-9 is a 36-kDa β-d-galactoside-binding protein comprised of two distinct carbohydrate recognition domains connected by a linker peptide in the N-and C-termini [9]. The galectin family is thought to regulate cell homeostasis and inflammation. Previous studies demonstrated that galectin-9 is distributed among tissues and induces various biological reactions such as cell aggregation, adhesion, chemoattraction, activation, and apoptosis [10]. Galectin-9 regulates the Th1/Th17 cell ratio to balance the immune response, thus playing a role in inflammatory diseases, and regulates T-cell immunity in chronic hepatitis C virus infection [11, 12]. In addition, galectin-9 expression was reported to be significantly elevated in the serum and lesional skin of patients with SSc, it was also considered to contribute to the Th immune balance in the lesional skin of SSc [13].

However, the role of galectin-9 in the pulmonary fibrosis of SSc remains unknown. In the present study, the expression level of galectin-9 in the lungs of patients with fibrosis was evaluated. Moreover, the effect of galectin-9 on fibrotic markers of mouse lung fibroblast cells and lung tissues was assessed in vitro and in vivo*.*

## Material and methods

### Patients and galectin-9 detection

Serum samples were obtained from 26 patients with SSc and 26 healthy controls. These samples were frozen at − 80 °C until analysis. Human galectin-9 levels were measured at the Inflammation Core Facility of the Institute of Biomedical Sciences, Academia Sinica, using their multiplex assay to measure cytokine/chemokine levels in patient sera. The level of the mediator was determined by a Bio-Plex 200 analyzer, which is a dual-laser, flow-based, sorting and detection platform. Additional information on the system can be found at the manufacturer’s website (https://www.ibms.sinica.edu.tw/inflammation_core_facility/page/s-a.html).

The mouse galectin-9 levels were detected by an enzyme-linked immunoassay kit from Elabscience (Houston, TX, USA) according to the manufacturer instructions.

### Mice

The Lgals9 knockout (KO) mice (strain B6(FVB)-Lgals9^tm1.1cfg^/Mmucd) were established by Dr. Jim Paulson. The commercial source can purchase from Mutant Mouse Resource and Research Center (MMRRC). Detailed genetic information of Lgals9 KO mice can be found on the CFG functional glycomics gateway site (http://www.functionalglycomics.org/static/consortium/resources/resourcecoref.shtml) or MMRRC (Citation ID: MMRRC_031952-UCD). C57BL/6 J mice were suggested as the wild-type (WT) controls per the MMRRC recommendation. Ten-to-twelve-week-old male mice were used for the experiments. The mice were obtained and bred in Taiwan National Laboratory Animal Center and National Applied Research Laboratories (NARLabs, Taipei, Taiwan), and housed according to the Principles of Laboratory Animal Care. The procedures for animal care and handling were approved by the Animal Committee of China Medical University. There were six to eight mice per group.

### Cell culture

Primary lung fibroblast cells were cultured from 8-week-old mice. In brief, the lungs of Lgals9 WT and KO mice were removed, added to Dulbecco’s modified Eagle’s medium containing 10% fetal bovine serum, and triturated using trypsin. Dissociated cells were then plated in 10-cm dishes and cultured for 1–2 weeks. After culturing, 3 × 10^5^ cells were seeded into 6-cm dishes and incubated with 40, 20, 10, 5, and 1 ng/ml recombinant murine TGF-β (PeproTech, Rocky Hill, NJ, USA) for 24 h, since TGF-β is a well-established primary mediator driving fibrogenesis [14]. The cells were then collected for RNA and protein extraction.

### Bleomycin-induced murine model of lung fibrosis and respiratory resistance

Bleomycin has been found to induce DNA strand breaks and oxidative stress, resulting in direct injury to the cell [15]. Subsequently, cell death occurs through either necrosis or apoptosis, accompanied by inflammation and fibrosis. To induce pulmonary fibrosis, bleomycin (Cayman Chemical, Ann Arbor, MI, USA) was applied intratracheally at 80 μg in a total volume of 20 μl to the mice twice a week, and the mice were sacrificed 4 weeks later. This dose was previously confirmed in mice for intratracheal delivery [16]. For intratracheal injection, the mice were placed in supine position on the operating field and the trachea was exposed with an otoscope. The bleomycin solution was injected into the trachea directly with a syringe through a 25-gauge needle. The lung tissues were isolated for further analyses. Airway responsiveness was expressed using the “enhanced pause” (Penh) as a parameter of altered airway function [17]. Penh is an empirical parameter that reflects changes in the box flow waveform from both inspiration and expiration. To measure respiratory system resistance, the mice were subjected to whole-body plethysmography for Penh recording (DSI Buxco, St. Paul, MN, USA).

### Histopathology and immunofluorescent staining

The lung tissues were fixed in 10% neutral-buffered formalin and embedded in paraffin. The tissues were cut into 5-μm sections and placed on slides, followed by staining with hematoxylin and eosin (H&E) and Masson’s trichrome (Leica Biosystems, Wetzlar, Germany). For immunofluorescent staining, 16-μm sections from O.C.T. (a tissue freezing medium)-embedded frozen tissues were blocked with 5% bovine serum albumin, incubated at room temperature with anti-alpha-smooth muscle actin (α-SMA) antibody (GeneTex, Irvine, CA, USA) overnight at 4 °C, and then incubated with Texas Red-conjugated secondary antibody (GeneTex) for 1 h at room temperature. The nuclei were stained with DAPI for 5 min at room temperature, and images were acquired with a fluorescence microscope (Olympus, Tokyo, Japan).

### RNA extraction and reverse transcription-quantitative polymerase chain reaction (qPCR)

Total RNA of the lung tissues and fibroblast cells was isolated using an RNeasy mini Kit (Qiagen, Hilden, Germany), and cDNA was synthesized using a High-Capacity cDNA Reverse Transcriptase Kit (Applied Biosystems, Foster City, CA, USA) according to the manufacturers’ instructions. *ACTA2, COL1A1, CTGF*, and *ET-1* transcript levels were then measured by qPCR using the cDNA as a template on a StepOne Plus system (Applied Biosystems) with universal probes (Roche, Basel, Switzerland) and the specific primer pairs listed in Table 1. The threshold cycle number (Ct) was calculated for each gene and normalized to that of glyceraldehyde 3-phosphate dehydrogenase (*GAPDH*). The ΔCt values for each gene are presented as relative fold-induction.

**Table 1:** Sequences of qPCR primers

Gene	Primer	Sequence 5′ – 3′	Probe no.
ACTA2	Forward	5′- CCAGCACCATGAAGATCAAG − 3′	58
ACTA2	Reverse	5′- TCCACATCTGCTGGAAGGTA − 3′	
COL1A1	Forward	5′- CATGTTCAGCTTTGTGGACCT − 3′	15
COL1A1	Reverse	5′- GCAGCTGACTTCAGGGATGT − 3′	
CTGF	Forward	5′- TGACCTGGAGGAAAACATTAAGA − 3′	71
CTGF	Reverse	5′- AGCCCTGTATGTCTTCACACTG − 3′	
ET-1	Forward	5′- TCCTTGATGGACAAGGAGTGT − 3′	29
ET-1	Reverse	5′- CCCAATCCATACGGTACGAC − 3′	
GAPDH	Forward	5′- GCCAAAAGGGTCATCATCTC − 3′	29
GAPDH	Reverse	5′- CACACCCATCACAAACATGG −3′	

### Western blotting analysis

The cells were washed twice with cold phosphate-buffered saline and lysed with RIPA lysis buffer (50 mM Tris-HCl pH 7.4, 150 mM NaCl, 1% NP40, 0.25% Na-deoxycholate, 1 mM PMSF) supplemented with a protease and phosphatase inhibitor cocktail (Roche). The protein concentrations of the cell lysis extracts were measured using the Bradford protein assay (Bio-Rad, Hercules, CA, USA) and equalized with the extraction reagent. Equal amounts of the proteins were loaded and subjected to sodium dodecyl sulfate-polyacrylamide gel electrophoresis, transferred onto 0.2-μm polyvinylidene fluoride membranes (Millipore, Billerica, MA, USA), and stained with appropriate antibodies (CTGF, ET-1, αSMA, and beta-actin: GeneTex, Irvine, CA, USA); Smad2, phospho-Smad2, Smad3, phospho-Smad3, Smad2/3, p-AKT, AKT, p- mitogen-activated protein kinase (MAPK), MAPK, p-c-Jun N-terminal kinase (JNK), and JNK: Cell Signaling Technologies, Danvers, MA, USA). The membranes were incubated with a 1:5000–10,000 dilution of horseradish peroxidase-conjugated anti-mouse or anti-rabbit secondary antibody (Cell Signaling) at room temperature for 2 h. Membranes were developed using the ECL system (ThermoFisher Scientific, Waltham, MA, USA) according to the manufacturer’s protocols. The reaction was visualized by chemiluminescence using an ImageQuant LAS4000 mini system (GE Healthcare, Little Chalfont, UK). Band intensity was quantified with ImageJ software (National Institutes of Health, Bethesda, MD, USA) and protein levels were normalized by beta-actin. In the graphs, the relative value compared with the control group is expressed as mean ± SD in arbitrary units.

### Statistical analysis

The data of galectin-9 expression in human and mouse sera were analyzed by the Mann-Whitney test and Student’s *t*-tests. Pearson’s coefficient analysis was used to analyze the correlation between the forced vital capacity (FVC) or diffusion capacity (DLCO) and galectin-9 expression level in the sera of SSc patients. The other data were analyzed using Student’s *t*-tests. A *p* value < 0.05 was considered statistically significant.

## Results

### Galectin-9 levels are increased in the serum of SSc patients

**Fig. 1:**
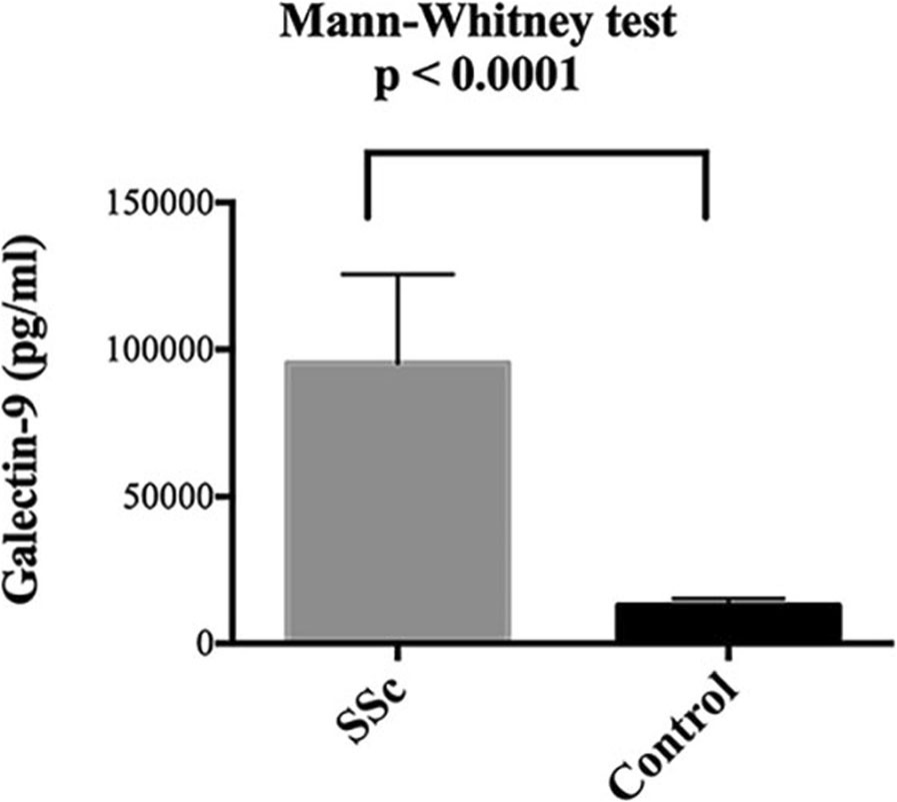
Clinical level of galectin-9 in the serum of patients with SSc. Serum galectin-9 levels were measured by a Bio-Plex assay and were compared to those of healthy controls

To investigate the contribution of galectin-9 to SSc, the concentration of galectin-9 in the serum was determined by bio-plex immunoassay. Galectin-9 levels were significantly higher (9-fold) in patients with SSc compared to those of healthy controls *(p* < 0.0001, Fig. 1). This result indicates that galectin-9 might be involved in the pathogenesis of SSc. We also examined the association of serum galectin-9 levels with clinical pulmonary function tests, including FVC and DLCO. We used Pearson’s correlation analysis to analyze the relationship between FVC or DLCO and galectin-9 expression levels in the serum of SSc patients, demonstrating a strong negative correlation with FVC but a weaker correlation with DLCO: FVC vs. galectin-9: r = − 0.737; DLCO vs. galectin-9: r = − 0.446 (mean FVC: 71.9 ± 14.3%, mean DLCO: 52.2 ± 19%, mean galectin-9 level: 31153 ± 18,832 pg/ml).

### Galectin-9 deficiency attenuated bleomycin-induced lung fibrosis

**Fig. 2:**
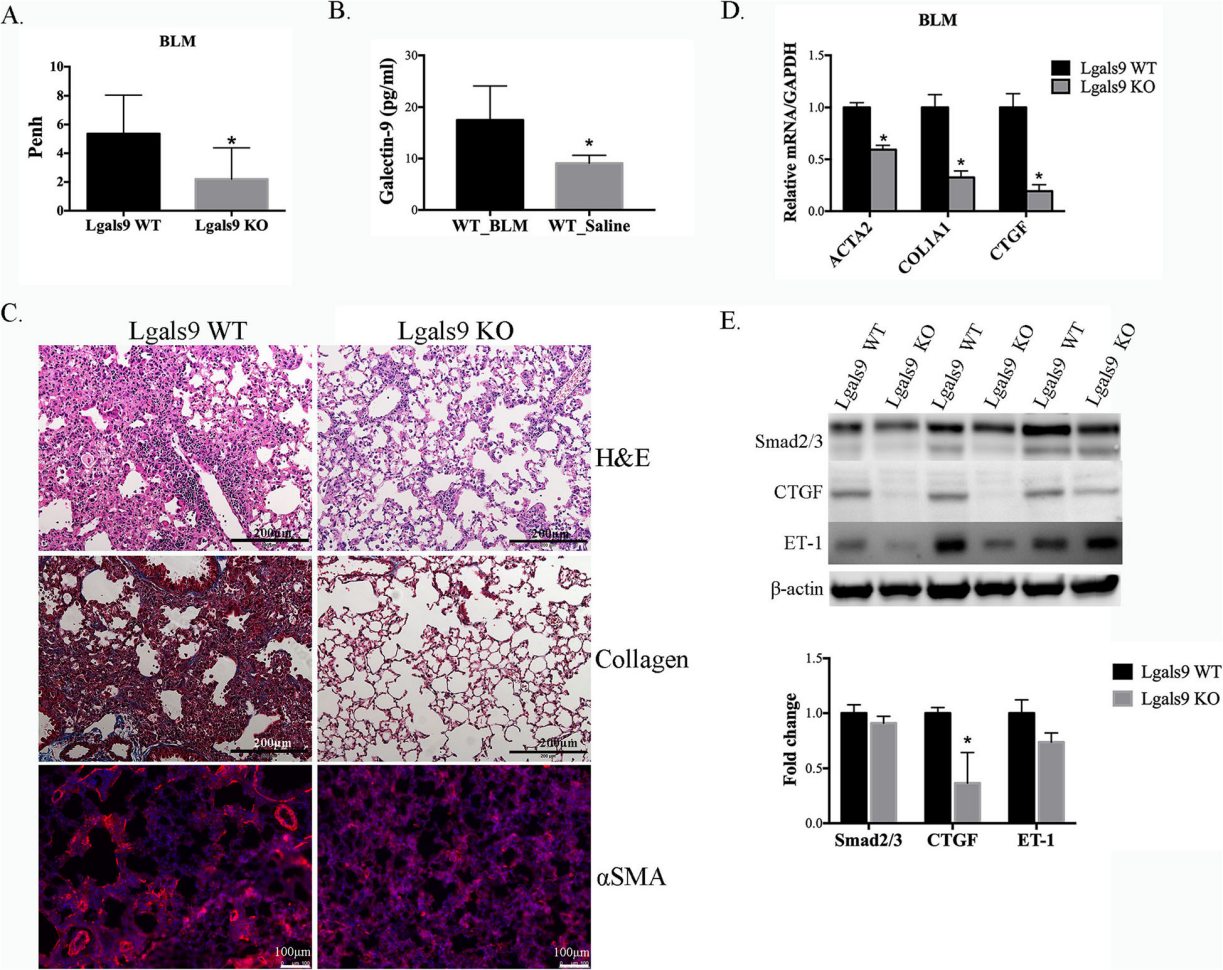
Effect of galectin-9 on bleomycin-induced lung fibrosis. **a** Analysis of Penh in galectin-9 wild-type (WT) mice and knockout (KO) mice. *p < 0.05. **b** Serum galectin-9 levels of bleomycin (BLM)- and saline-treated WT mice. *p < 0.05. **c** Lung tissue sections stained by hematoxylin and eosin (H&E, upper) and Masson’s trichrome stain for collagen I (middle), and immunofluorescence for αSMA (red) and DAPI nuclear staining (blue) (lower). Scale bar: 200 μm (upper and middle) and 100 μm (lower). **d** *ACTA2*, *CTGF*, and *ET-1* mRNA levels in the lung tissues of galectin-9 WT and KO mice treated with bleomycin for 4 weeks assessed by qPCR. The relative values are presented compared with those of the WT group. * *p* < 0.05. **e** Western blot analysis of Smad2/3, CTGF, ET-1, and β-actin. Protein expression levels were normalized to β-actin expression and compared with those of the WT group. Data are shown at the mean ± SD, *n* = 3. *p < 0.05

Fibrosis is the major characteristic of SSc, and bleomycin has been shown to cause pulmonary fibrosis [18]. Therefore, we next investigated the effect of galectin-9 on pulmonary fibrosis induced by bleomycin in mice. Bleomycin was administered into the lung via the intratracheal route at 80 μg twice weekly for 4 weeks to induce fibrosis. Before sacrifice, the mice were subjected to whole-body plethysmography to detect the level of Penh. Galectin-9 KO mice showed lower levels of Penh (*p* < 0.041 Fig. 2a) than those of WT mice. Thus, respiratory inflammation was ameliorated in mice with galectin-9 deficiency. In addition, the serum galectin-9 level was significantly increased in bleomycin-treated WT mice compared with that of saline-treated WT mice (*p* = 0.0248, Fig. 2b). In the lung tissues, bleomycin induced fibrosis development to a greater extent in WT mice than in galectin-9 KO mice according to H&E staining (Fig. 2c, upper). Collagen fiber production was also higher in WT tissues according to Masson trichrome staining (Fig. 2c, middle). Moreover, the intensity of αSMA staining, which plays an important role in fibrogenesis, was higher in WT than in galectin-9 KO mouse lung tissues (Fig. 2c, below). Consistently, qPCR using the WT tissues revealed significantly higher mRNA expression levels of *ACTA2*, *COL1A1*, and *CTGF* (Fig. 2d). Furthermore, the levels of the fibrotic proteins Smad2/3, CTGF, and ET-1 were determined by western blotting. The CTGF expression level in galectin-9 WT mice was significantly higher (*p* < 0.05) than that of the galectin-9 KO mouse tissues. Although galectin-9 KO tissues exhibited slightly lower expression levels of Smad2/3 and ET-1, the differences were not significantly significant between the two groups (Fig. 2e). Collectively, these results indicate that galectin-9 expression is involved in fibrosis progression through TGF-β-activated ACTA2, CTGF, and ET-1. In contrast, fibrosis was improved when galectin-9 was deficient.

### Lack of galectin-9 decreased the progression of fibrosis by TGF-β

**Fig. 3:**
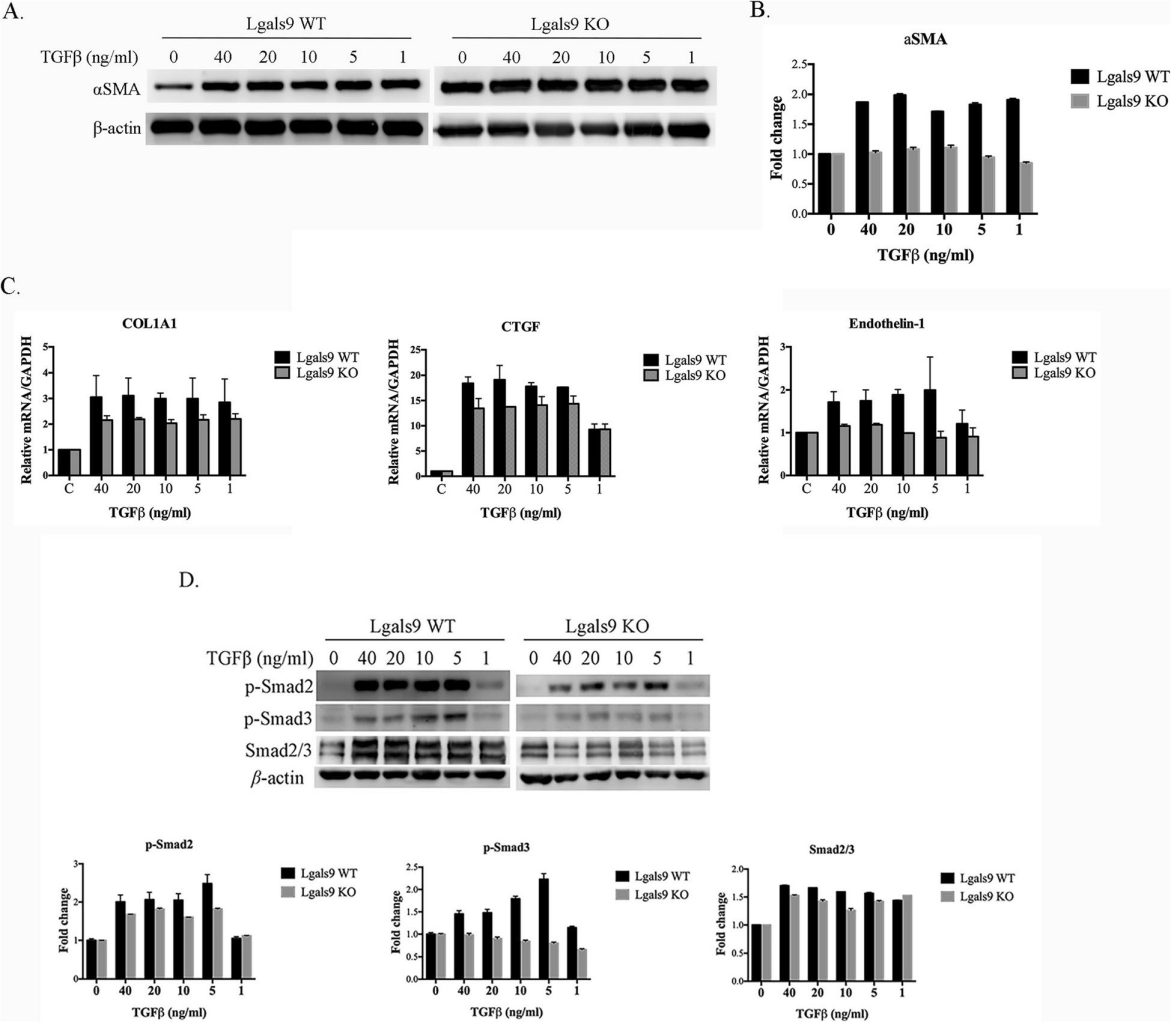
Effect of galectin-9 on fibrotic markers and the TGF-β signaling pathway in lung fibroblast cells. **a** αSMA and β-actin expression determined by immunoblotting in primary lung fibroblast cells of galectin-9 wild-type (WT) and knockout (KO) mice treated with the indicated concentrations of TGF-β for 24 h. **b** Protein expression levels were normalized to the level of β-actin. The relative fold changes in expression levels of the galectin-9 WT and KO groups were respectively compared with the levels of TGF-β untreated cells (0 ng/ml). Data are shown as the mean ± SD, n = 3. **c** Primary lung fibroblast cells of galectin-9 WT and KO mice treated with the indicated concentrations of TGFβ for 24 h. The mRNA levels of *COL1A1*, *CTGF*, and *ET-1* were determined by qPCR. The relative value was compared to that of the control group. **d** Western blot analysis of p-Smad2, p-Smad3, Smad2/3, and β-actin. Protein expression levels were normalized to the level of β-actin. The relative fold changes in expression levels of the galectin-9 WT and KO groups were respectively compared with those of TGF-β untreated cells (0 ng/ml). Data are shown as the mean ± SD, *n* = 3

To examine the difference in galectin-9 WT and KO mice in the development of TGF-β-activated fibrosis, we examined primary fibroblast cells from both types of mice. TGF-β induced αSMA expression in a dose-dependent manner in WT fibroblast cells. In addition, the fold-change in TGF-β-induced αSMA protein expression was markedly higher in WT cells than in KO cells respectively compared with that of control cells not treated with TGF-β (Fig. 3a and b). Similar effects on *COL1A1*, *CTGF*, and *ET-1* were observed by qPCR (Fig. 3c). Finally, we evaluated the Smad-dependent pathway induced by TGF-β. TGF-β induced transcriptional regulation by phosphorylating the Smad2 and Smad3 proteins, followed by an interaction with Smad4. As shown in Fig. 3d, TGF-β significantly induced Smad2 and Smad3 phosphorylation in WT tissues. Cells from the mice defective in galectin-9 showed a reduced response to TGF-β. These findings indicate that lack of galectin-9 in fibroblasts suppresses TGF-β-related reactions.

### Galectin-9 deficiency affects fibrosis progression via a Smad-independent pathway in vivo and in vitro

**Fig. 4:**
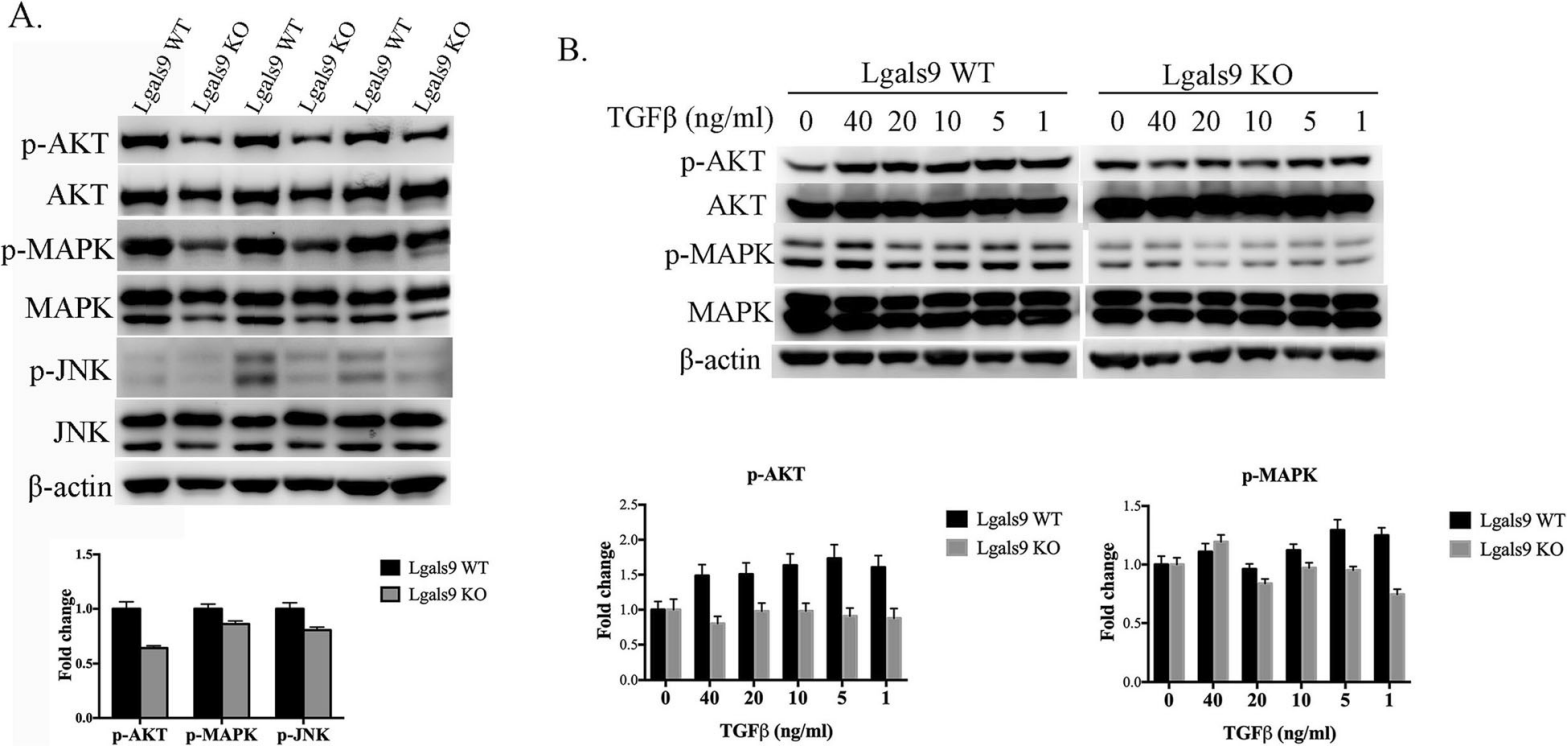
Effect of galectin-9 on the AKT, MAPK, and JNK pathways in vivo and in vitro. **a** Protein and phosphorylation levels in the lung tissues of galectin-9 wild-type (WT) and knockout (KO) mice treated with bleomycin for 4 weeks analyzed by western blotting for p-AKT, AKT, p-MAPK, MAPK, p-JNK, JNK, and β-actin. Protein and phosphorylation levels were normalized to the level of β-actin. The relative fold change was compared with the WT group. Data are shown as the mean ± SD, n = 3. **b** Western blotting for p-AKT, AKT, p-MAPK, MAPK, and β-actin protein expression in primary lung fibroblast cells treated with the indicated concentrations of TGF-β for 24 h. Protein expression levels were normalized to that of β-actin. The relative fold change in expression levels of the galectin-9 WT and KO groups was respectively compared with that of TGF-β untreated cells. Data are shown as the mean ± SD, *n* = 3

TGF-β has been recognized as a central mediator of tissue fibrosis. The major mechanism involves delivery of the signal via Smad molecules through the Smad-dependent pathway. However, the MAPK/extracellular related kinase (ERK), P38, c-JNK, nuclear factor-κB, and phosphatidylinositol 3-kinase (PI3K) signaling pathways have also been implicated in the fibrosis responses induced by TGF-β [19]. To evaluate the effect of galectin-9 deficiency on the Smad-independent pathways of fibrosis, the lung tissues of bleomycin-treated galectin-9 WT and KO mice were isolated and the proteins were extracted. The levels of phosphorylated AKT, MAPK, and JNK proteins were higher in galectin-9 WT than those of KO mice (Fig. 4a). Consistent results were observed in mouse primary lung fibroblast cells; the change in phosphate AKT and MAPK by TGF-β showed a greater increase following administration of galectin-9 (Fig. 4b). These findings indicate that galectin-9 promotes signaling pathways that cause fibrosis via TGF-β.

## Discussion

SSc is a chronic systemic autoimmune disease characterized as a multisystemic connective tissue disease caused by inflammation and fibrosis on the skin or internal organs [1]. The major pathogenic factors responsible for the various clinical features are vascular injury, fibrosis, and immune activation [20]. Studies have also indicated that inflammatory cytokines induce endothelial cell injury leading to vascular damage [21]. Expression of these cytokines can cause fibrosis by inducing excess extracellular matrix synthesis and collagen accumulation. The vascular injury is mediated by ET-1 and TGF-β activation, which contribute to fibroblast activation and myofibroblast transdifferentiation [22]. Myofibroblasts expressing αSMA are the primary moderators of fibrosis caused by the excess deposition of extracellular matrix [23]. TGF-β induces αSMA expression via the Smad signaling pathways, and can induce ET-1 and CTGF production through Smad-dependent or Smad-independent signaling pathways, including the MAPK/ERK, TAK1/JNK, and PI3K/AKT pathways, which are also activated by TGF-β [19].

However, the factors contributing to the initiation of the pathogenesis of SSc remain unclear. The administration of bleomycin is a widely used method to induce pulmonary fibrosis in animal models. Bleomycin induces DNA strand breaks and oxidative stress to directly injure the cell. Subsequently, cell death occurs through either necrosis or apoptosis with consequent development of inflammation and fibrosis [16]. We found that the levels of fibrotic markers such as αSMA, CTGF, and collagen were significantly higher in the bleomycin-induced lung fibrosis model than controls, suggesting a suitable model for assessing the role of galectin-9 in the lung fibrosis accompanying SSc.

Galectin-9 is a β-galactoside lectin with two carbohydrate recognition domains at the N- and C-termini connected by a linker peptide. Galectin-9 is encoded by *LGALS9*, which is widely distributed among tissues, and is predominantly expressed by activated endothelial cells, interferon (IFN)-stimulated fibroblasts, and innate or adaptive immune cells [24]. Galectin-9 is highly expressed in the liver and circulation in patients with chronic liver diseases, and higher serum galectin-9 levels are related to liver fibrosis progression [25]. The clinical association of serum galectin-9 levels with SSc has also been investigated in patients [13]. Similar results were observed in our study, with higher expression of galectin-9 detected in the serum of patients with SSc. Thus, galectin-9 might be useful as a biomarker of fibrosis in SSc.

TGF-β is known to induce fibroblast growth and collagen synthesis. Enhanced TGF-β signaling has been observed in SSc fibroblasts in vivo and in vitro [26]. Upon TGF-β binding to the TGF-β receptors TβRI and TβRI, the receptors are phosphorylated and transiently associate with Smads (Smad2 and Smad3). Receptor-activated Smads are phosphorylated and then form a heterooligomeric complex with Smad4 for translocation to the nucleus to induce target gene transcription [27]. TGF-β regulates expression of the αSMA gene (*ACTA2*) via Smad3 activation in myofibroblast differentiation [22]. Moreover, increased Smad2 and Smad3 phosphorylation by TGF-β was observed in scleroderma fibroblasts [8]. TGF-β is mainly derived by the peripheral naïve T cells that differentiate into Foxp3^+^ iTreg cells. Smad3 enhances Foxp3 expression, and activation of Smad3 and Foxp3 ensure the stable formation of iTreg cells. Previous studies showed that mice lacking galectin-9 exhibited decreased Foxp3 expression levels, and *Lgals9*^−/−^ T cells were defective in Foxp3 expression. Galectin-9 promotes iTreg differentiation via the TGF-β-induced phosphorylation of Smad2/3, MAPK/ERK, and complex formation of Smad2/3 with Smad4 [28, 29]. These data demonstrate that galectin-9 participates in further regulation through both Smad-dependent and -independent pathways. In line with the results of the present study, normal expression of galectin-9 results in high activation of Smad2/3 and significant fold-induction of ACTA2, COL1A1, CTGF, and ET-1 expression. Galectin-9 deficiency reduced the production of these fibrotic molecules. TGF-β activated the Smad-independent MAPK/ERK, TAK1/JNK, and PI3K/AKT pathways to mediate tissue fibrosis. We also found that TGF-β induced higher levels of phosphorylation of AKT, MAPK, and JNK in both the lung tissues and lung fibroblast cells of WT mice. In contrast, lack of galectin-9 ameliorated the progression of fibrosis by TGF-β.

In terms of immunity, CD4^+^ T cell activation and infiltration in the skin and internal organs occurs in the early phase of SSc. Activated T cells, B cells, and non-specific inflammatory cells infiltrate various tissues to cause damage to the fibroblasts and endothelial cells by inducing several mediators [30]. The balance between Th1 and Th2 cytokines is altered in tissue injury. T cells polarized toward the Th2 pattern, which secrete abundant IL-4, IL-5, and IL-13, contribute to formation of a pro-fibrotic environment [31]. In contrast, the Th1 cytokine IFNγ is associated with anti-fibrotic effects. Lower levels of IFNγ in the blood and a deficiency of IFNγ production in peripheral mononuclear cells and bronchoalveolar lavage cells have been investigated in patients with SSc. Studies of Th2/Th17-skewed immune polarization in SSc indicated that IL-33 production induced the skin-localized transdifferentiation of Tregs into Th2-like cells [32]. The immunomodulatory effect of galectin-9 has been studied based on its interaction with the glycoprotein ligand TIM-3. TIM-3 is highly expressed on Th1 cells and by activated CD4^+^ cells in humans [33]. In addition, activated CD4^+^ T cells secreted IFNγ, IL-17, IL-2, and IL-6, but not IL-10, IL-4, or tumor necrosis factor-α. In mice, galectin-9 also mediated the decrease in Th1 and Th17 cell infiltration, which was associated with the downregulation of CXCL9, CXCL10, and CCL20 expression [11]. Elevated galectin-9 expression was also observed in SSc dermal fibroblasts in vivo and in vitro. Importantly, galectin-9 overproduction can suppress IFNγ expression by CD4^+^ T cells in *Fli1*^+/−^ dermal fibroblasts. Bleomycin-induced skin fibrosis was attenuated by galectin-9 deficiency and increased IFNγ production [11, 13]. These findings were similar with our present results, suggesting that the loss of galectin-9 significantly reduces fibrosis and plays a role in the balance of Th1/Th2 immunity.

## Conclusion

We investigated the role of galectin-9 expression in the serum of patients with SSc. We found increased expression levels of collagen and αSMA in lung sections of mice induced to develop lung fibrosis with bleomycin as an SSc animal model. In addition, higher mRNA expression levels of *ACTA2, COL1A1*, and *CTGF* were observed in the bleomycin-treated lung specimens of mice expressing normal levels of galectin-9 compared to those with galectin-9 deficiency. Similar results for the protein expression of Smad2/3, CTGF, and ET-1 were detected under galectin-9 deficiency. Activation of TGF-β signaling was shown to up-regulate the expression of downstream fibrotic markers in fibroblast cells expressing galectin-9. Moreover, the MAPK/ERK, TAK1/JNK, and PI3K/AKT signaling pathways were influenced by the presence of galectin-9 in lung tissues and fibroblast cells. These results suggest that galectin-9 acts as a potent mediator of fibrosis progression.

In conclusion, our findings suggest an important role for galectin-9 as a mediator of the TGF-β-induced progression of lung fibroblast cells into fibrosis. Data from the bleomycin-induced lung fibrosis model showed consistent results. We also demonstrated that galectin-9 promotes fibrosis development via the entire TGF-β signaling pathway. Therefore, galectin-9 is a potential biomarker that may also serve as a novel target for therapeutic intervention in SSc.

## Data Availability

The data that support the findings of this study are available from the corresponding author upon reasonable request.
